# Prognostic value of the common tumour-infiltrating lymphocyte subtypes for patients with non-small cell lung cancer: A meta-analysis

**DOI:** 10.1371/journal.pone.0242173

**Published:** 2020-11-10

**Authors:** Benchao Chen, Heng Li, Chao Liu, Xudong Xiang, Shuting Wang, Anhao Wu, Yan Shen, Gaofeng Li

**Affiliations:** Department of Thoracic Surgery, The Third Affiliated Hospital of Kunming Medical University (Tumor Hospital of Yunnan Province), Kunming, Yunnan, China; Taichung Veterans General Hospital, TAIWAN

## Abstract

**Background:**

Many previous studies have revealed that tumour-infiltrating lymphocytes (TILs) are significantly associated with prognosis in various tumours. However, this finding remains controversial in non-small cell lung cancer (NSCLC). We performed this meta-analysis systematically to evaluate the prognostic value of TILs in NSCLC.

**Methods:**

The references were collected by searching the PubMed, EMBASE and Web of Science databases. The pooled hazard ratios (HRs) with 95% confidence intervals (CIs) were summarized using random or fixed effects models to evaluate the association between TILs and NSCLC survival outcomes.

**Results:**

A total of 45 interrelated studies were eligible that included 11,448 patients. Pooled analysis showed that a high density of TILs indicated a better overall survival (HR = 0.80, 0.70–0.89) and progression-free survival (HR = 0.73, 0.61–0.85) for patients with NSCLC; a high density of CD3+ TILs in the tumour nest indicated a better overall survival (HR = 0.84, 0.69–0.99) and disease-specific survival (HR = 0.57, 0.34–0.80); a high density of CD4+ TILs in the tumor nest indicated a favourable overall survival (HR = 0.86, 0.76–0.96); a high density of CD8+ TILs indicated a favourable overall survival (HR = 0.995, 0.99–1.0), progression-free survival (HR = 0.52, 0.34–0.71), disease-free survival (HR = 0.64, 0.43–0.85), relapse/recurrence-free survival (HR = 0.42, 0.18–0.67) and disease-specific survival (HR = 0.56, 0.35–0.78); and a high density of CD20+ TILs in the tumour nest indicated a favourable overall survival (HR = 0.65, 0.36–0.94). However, a high density of Foxp3+ TILs in the tumour stroma indicated a worse relapse/recurrence-free survival (HR = 1.90, 1.05–2.76) in NSCLC.

**Conclusions:**

Our meta-analysis confirmed that high densities of TILs, CD3+TILs, CD4+TILs, CD8+TILs and CD20+TILs in the tumour nest are favourable prognostic biomarkers for patients with NSCLC, and Foxp3+TILs in the tumour stroma are a poor prognostic biomarker.

## Introduction

Lung cancer is one of the most common cancers worldwide and has the highest morbidity and mortality of all malignant tumours [1]. The major histologic subtype of lung cancer is non-small cell lung cancer (NSCLC 85%) [2]. The only chance for clinical cure is surgical treatment in the early stages of NSCLC, but on account of the lack of early adequate screening, most patients with NSCLC are diagnosed at an advanced stage [3]. Due to the lack of sensitive measures to evaluate the prognosis of NSCLC, treatment methods cannot be enacted in a timely manner, which leads to 60% of patients with locally advanced NSCLC experiencing relapse after therapy or dying of metastasis [4]. Although multidisciplinary synthetic therapy has been used to treat patients with NSCLC, the survival rate of patients with NSCLC has barely improved over recent years [5]. At present, the prognosis of patients with NSCLC was forecasted on the basis of TNM staging based on histopathology or imageology, and this method has been proved that it is not precise by growing evidence [6]. Therefore, a new accurately and effectively biological marker is needed to evaluate the prognosis of NSCLC.

Much attention has been paid to the role of the immune response in NSCLC in the past decades [7, 8]. The NSCLC microenvironment has been a focus in the exploration of an accurate prognostic biomarker. TILs are a local histopathological reflection of the host’s immune response against cancer cells. Currently, TILs have gained increasing attention in the treatment and prognosis of NSCLC [9–11]. TILs can be divided into two groups according to the position of infiltration: lymphocytes within the tumour nest (TN) and lymphocytes in the tumour stroma (TS). The density of TILs was detected by immunohistochemistry (IHC) and/or hematoxylin & eosin (H&E) staining [12]. The density of TILs was calculated according to the percentage or the cell population of the tumour tissue area that stained positively, but there is no standardized cut-off value for evaluating the density of TILs. Some studies have indicated a distinct correlation between different TIL densities or positions and different prognostic values.

TILs not only include T cells but also involve B cells, dendritic cells (DCs), natural killer (NK) cells and other types of immune cells [13]. Many articles have shown that a high density of TILs of different subtypes are a good prognostic biomarker for patients with NSCLC [14–18]. However, other studies have also demonstrated that a high density of TILs of different subtypes is an inferior prognostic biomarker for patients with NSCLC [17, 19, 20]. Therefore, previous studies remain controversial about the prognostic value of the common TIL subtypes in NSCLC. The conflicting reports on the prognostic value of TILs in NSCLC may be due to the position of infiltration and histologic subtypes, different patient populations and different TIL evaluation systems. Thus, it is crucial to understand the prognostic value of the different TIL phenotypes.

Articles that have been published about the prognostic value of TIL, CD3+, CD4+, CD8+, FoxP3+, and CD20+ TIL subsets in NSCLC were systematically reviewed in our meta-analysis. We aim to include all correlational studies to assess the prognostic value of TILs and attempt to provide an accurate biomarker to guide prognosis and treatment for NSCLC in the future.

## Methods

### Search strategy

The PRISMA guidelines were followed for our meta-analysis (**S1 Table**) [21]. The literature was obtained by searching the PubMed, EMBASE and Web of Science databases from the initiation to July 28, 2020. The search strategy included the domain (“lung cancer"), the determinant ("Tumor-infiltrating cells", "TILs") and their synonyms. There was no limitation on the publication status, but we excluded the studies in languages other than English. All eligible studies were retrieved, and three researchers independently screened the titles and abstracts of all the reference lists of the reviews or studies based on the criteria. Differences between two authors were resolved by the third author’s opinion, and we included studies that received two votes.

### Inclusion criteria

Studies were eligible for inclusion according to the following criteria: (1) studies that reported the prognostic value of TILs, CD3+, CD4+, CD8+, CD20+ and FoxP3+ lymphocytes in NSCLC and analysed lymphocytes in the tumour nest (TN) or tumour stromal (TS); (2) studies that were published as original articles; (3) studies that provided adequate data to compute the HRs and 95% CIs; and (4) studies in which the prognostic value was investigated by survival analysis with either overall survival (OS), disease-free survival (DFS), progression-free survival (PFS), relapse/recurrence-free survival (RFS) or disease-specific survival (DSS). Animal studies, case reports, and commentaries were excluded.

### Data extraction

The data were independently extracted by three authors, which including the author and country, year of publication, sample size, tumour stage, histologic subtype, TIL locations, biomarker(s), scoring methods and therapy method, and finally the main outcomes, which included OS, DFS, PFS, RFS and DSS. The outcomes from univariate or multivariate Cox regression, HR and 95% CI, were used for analysis. If univariate and multivariate analysis outcomes were both mentioned in one study, only the multivariate analysis outcome was extracted. When these critical data were not mentioned in the article but the Kaplan-Meier curves were available, we extracted and digitized the data from the Kaplan-Meier curves by using the Engauge Digitizer software (http://digitizer.sourceforge.net/), and then the univariate HRs and 95% CIs were estimated by Excel programme files that were exploited by Jayne F Tierney teamwork [22]. When the HRs and 95% CIs were not mentioned and Kaplan-Meier curves were not available, we tried to send an e-mail to the corresponding authors of eligible articles to obtain the original data. The studies were excluded from the meta-analysis if we received nothing.

### Assessment of study quality

Three authors independently used the Quality In Prognosis Studies (QUIPS) tool to assess the risk of bias of all publications[23]. According to the criteria, every article was evaluated as low risk, moderate risk or high risk based on six different areas: study participation, study attrition, prognostic factor measurement, outcome measurement, study confounding, statistical analysis and reporting [23]. Differences were settled through discussion.

### Statistical analysis

Three authors independently pooled the HR and 95% CI from the original articles. HRs was used to describe the risk of events for a high density of TILs versus a low density of TILs. We considered the reciprocal of the HR if the study reported low TILs vs. high TILs. We observed that HR > 1 indicated a worse prognosis in patients with a high density of TILs and HR < 1 suggested a better prognosis. For the overall results, *p* < 0.05 was considered statistically significant. Statistical heterogeneity was assessed by calculating the *I*^*2*^ statistic [24]. The presence of heterogeneity was indicated by *I*^*2*^ > 50%, and a random-effects model (the DerSimonian-Laird method) was applied [25]; otherwise, a fixed-effects model (the Mantel–Haenszel method) was applied [26]. When heterogeneity was observed, either subgroup or sensitivity analysis was performed. The potential publication bias was estimated by Begg’s and Egger’s tests, where *p* < 0.05 indicates publication bias and *p* > 0.05 indicates no bias [27]. The meta-analysis and creation of the forest plots were performed in Stata15.0 software.

## Results

### Study selection and basic characteristics

**Fig 1:**
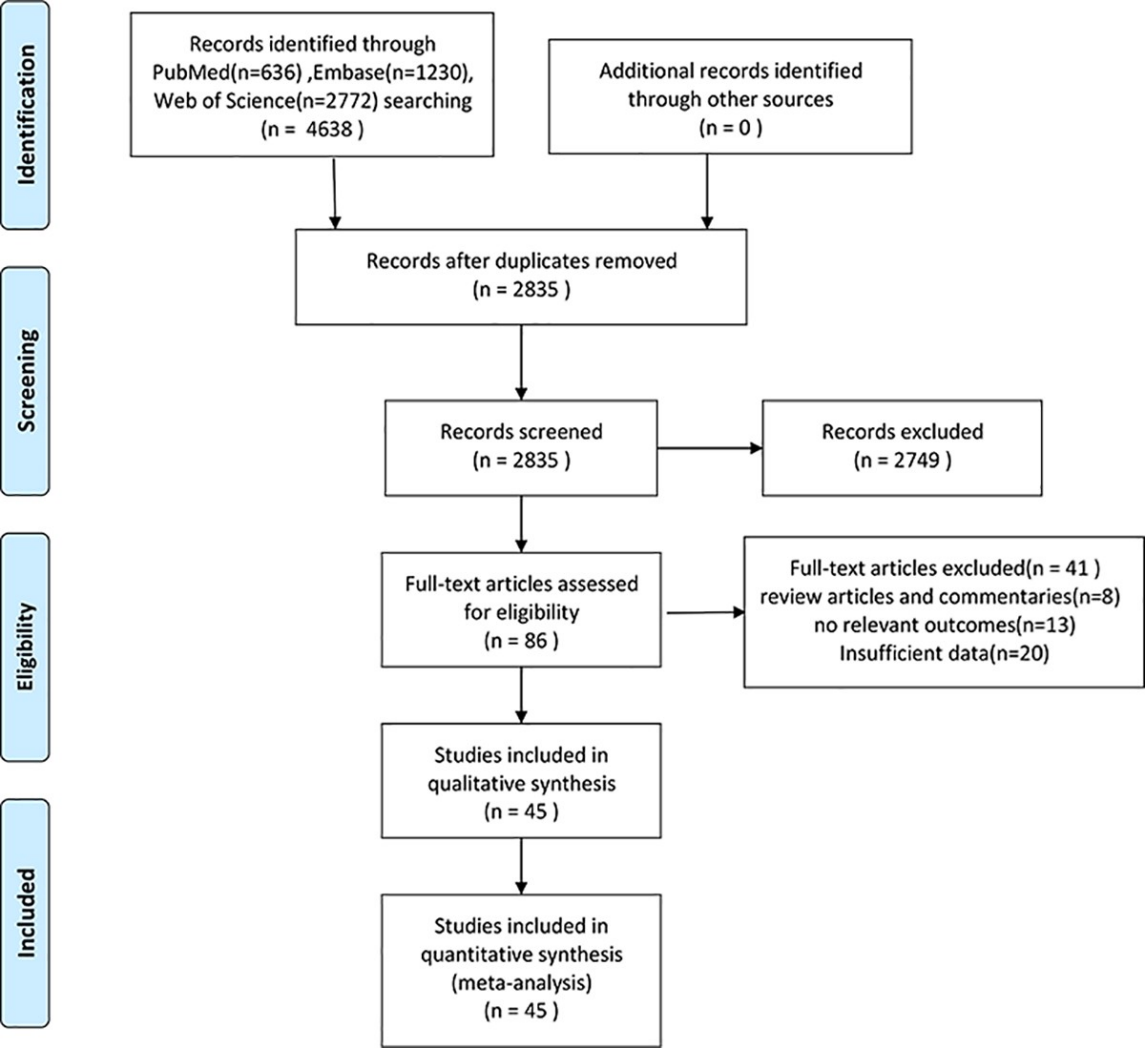
Flow diagram of study selection.

Using the search strategy described above, a total of 4638 original documents were obtained from databases, and approximately about 2835 studies remained after excluding duplicates. After screening the titles and abstracts of the 2835 publications, 2749 publications were not related to evaluating the prognostic role of TILs in lung cancer. Finally, we retained 86 eligible studies after screening the full text, among which 45 articles were included in our final analysis (**Fig 1**) [14–20, 28–65]. Forty-one articles were excluded for the following reasons: 8 articles were review articles and commentaries, 20 articles reported no hazard ratios or Kaplan-Meier curves, 5 articles only reported the HR in small cell lung cancer (SCLC), 2 articles reported that patients received neoadjuvant therapies before their research, and 6 articles did not report relevant outcomes. The main study characteristics of the 45 eligible articles are summarized in **Table 1**. All these articles were published from the initiation to July 28, 2020, and all of the patients were diagnosed with NSCLC by histopathology. Among these studies, six studies [28, 30, 35, 53, 54, 56] reported the prognostic value of TILs. Other studies have mainly paid close attention to the prognostic value of TIL subsets, such as CD3+, CD4+, CD8+, CD20+ and FoxP3+ lymphocyte subsets in the TN and/or TS of NSCLC. All studies investigated TILs by immunohistochemistry (IHC) and/or H&E staining of paraffin-embedded tissue. All tissues came from patients with NSCLC after surgery. In the remaining studies, OS was defined as the time from pathological diagnosis to the time of death or last follow-up, DFS was defined as the time from pathological diagnosis to the date of first recurrence and/or disease progression (regional or distant metastases) or last follow-up, PFS was defined as the time from pathological diagnosis to the date of progression, and RFS was defined as the time from pathological diagnosis to the date of recurrence. DSS was calculated from the date of pathological diagnosis to the date of death from cancer or last follow-up.

**Table 1:** Study characteristics of the 45 eligible articles.

study	year	country	NO. of samples	TILs location	tumor stage (I/II/III/IV)	histologic subtype	biomarkers	outcomes	method	therapy
Jeremy Goc[14]	2014	America	376	TN/TS	I-IV	NSCLC	CD8+	OS	IHC	surgery
Jiewei Chen[15]	2018	China	100	TN	I-IV	NSCLC	CD8+	OS/PFS	IHC	surgery
Yoichi Ohtaki[16]	2018	Japan	95	TN/TS	I-IV	NSCLC	CD4+/CD8+/Foxp3+	OS/RFS	IHC	surgery
Chuntao Tian[17]	2015	China	129	TN	I-III	NSCLC	CD3+/CD8+	OS	IHC	surgery
Zhangguo Hu[18]	2018	China	90	TN	I-IV	NSCLC	CD8+/CD45+/Foxp3+	OS/DFS	IHC	surgery
Zachary D. Horne, B.S.[28]	2011	America	273	TN	I	NSCLC	TIL	OS/RFS	IHC	surgery
Xiangjiao Meng[29]	2018	China	197	TN/TS	I-III	NSCLC	CD4+/CD8+/Foxp3+	OS	IHC	surgery
Wen Feng[30]	2016	China	320	TN	IIIA	NSCLC	TIL	OS	IHC	surgery
Tom Donnem[31]	2015	Norway	797	TS	I-IIIA	NSCLC	CD8+	OS/DFS/DSS	IHC	surgery
TAKEO HASEGAWA[32]	2014	Japan	67	TN/TS	I-IIIB	NSCLC	CD4+/CD8+/Foxp3+	OS	IHC	surgery
T. Kinoshita[33]	2016	Japan	218	TN	II-III	NSCLC	CD8+/CD20+/Fxp3+/Treg	OS	IHC	surgery
Souptik Barua[34]	2018	America	120	TN	I-III	NSCLC	CD4+/CD8+/CD68+/Foxp3+	OS	IHC	surgery
Satoshi Ikeda[35]	2005	Japan	83	TN	I-III	NSCLC	CD8+/TIL	OS	IHC	surgery
Rebecca P. Petersen[36]	2006	USA	64	TN	I	NSCLC	CD3+/Foxp3+	OS	IHC	surgery
Osamu Wakabayashi[37]	2003	Japan	178	TN/TS	I-IIIA	NSCLC	CD4+/CD8+	OS	IHC	surgery
Mehrdad Talebian Yazdi[38]	2015	Netherlands	197	TN/TS	I-IV	NSCLC	CD8+/HLA	OS	IHC	surgery
Marta Usó[39]	2016	Spain	84	TN/TS	I-IIIA	NSCLC	CD8+/Foxp3+	OS/PFS	IHC	surgery
Marius Ilie[40]	2011	France	632	TN	I-III	NSCLC	CD8+	OS	IHC	surgery
Marie-Caroline Dieu-Nosjean[41]	2008	France	74	TN	I-III	NSCLC	CD3+/CD20+/CD45+	OS/DSS/DFS	IHC	surgery
Kyuichi Kadota[42]	2015	America	331	TN	I-III	NSCLC	CD3+/CD4+/CD8+/CD20+/Foxp3+	OS	IHC	surgery
Khalid I. Al-Shibli[43]	2008	Norway	335	TN/TS	I-IIIA	NSCLC	CD4+/CD8+/CD20+	DSS	IHC	surgery
KHALID AL-SHIBLI[44]	2010	Norway	335	TN/TS	I-IIIA	NSCLC	CD3+/CD117+/CD138+	DSS	IHC	surgery
K Hiraoka[45]	2006	Japan	109	TS	I-III	NSCLC	CD4+/CD8+	OS	IHC	surgery
Hui Yang[46]	2018	China	178	TN	I-IV	NSCLC	CD8+	OS	IHC	surgery
Hiroyuki Tao[47]	2012	Japan	87	TN	I-III	NSCLC	Foxp3+	OS/RFS	IHC	surgery
Haiyue Wang[48]	2018	China	159	TS	I-III	NSCLC	CD8+	OS/PFS	IHC	surgery
Gian Kayser[49]	2012	Germany	232	TS	I-IV	NSCLC	CD3+/CD3+CD8+/CD4+CD25+	OS	IHC	surgery
Fuqiang Dai[50]	2010	China	99	TN/TS	I-IV	NSCLC	CD8+	OS	IHC	surgery
Feifei Teng[51]	2016	China	126	TS	I	NSCLC	CD8+/Foxp3+	OS/DFS	IHC	surgery
Fayc¸al Djenidi[52]	2015	France	101	TN/TS	I	NSCLC	CD3+/CD8+/CD103	OS/DFS	IHC	surgery
Enrico Ruffini[53]	2009	Italy	1290	TN	I-IIIA	NSCLC	TIL	OS	IHC	surgery
Eiki Kikuchi[19]	2007	Japan	161	TN/TS	I-IV	NSCLC	CD8+	OS	IHC	surgery
Dermot S. O’Callaghan[20]	2015	Australia	197	TN/TS	I-IIIA	NSCLC	CD3+/CD8+/Foxp3+	OS	IHC	surgery
Mariam Gachechiladze[54]	2020	Czech	1205	TN	I-III	NSCLC	CD3+/CD8+/TIL	OS/PFS	H&E	surgery
Fumihiko Kinoshita[55]	2020	Japan	203	TN	IA	NSCLC	CD8+/Foxp3+	OS/DFS	IHC	surgery
Ahrong Kim[56]	2019	Korea	146	TN	I-IV	NSCLC	TIL	OS/PFS	H&E	surgery
Lu Chen[57]	2019	China	354	TN/TS	I-IV	NSCLC	CD8+	OS	IHC	surgery
Yoshinori Handa[58]	2020	Japan	126	TN/TS	I	NSCLC	CD4+/CD8+/Foxp3+	RFS	IHC	surgery
Arik Bernard Schulze[59]	2020	Germany	294	TN	I-III	NSCLC	CD4+/CD8+/Foxp3+	OS/PFS	IHC	surgery
Kei Suzuki[60]	2012	USA	478	TS	I	NSCLC	Foxp3+	RFS	IHC	surgery
Germán Corredor[12]	2019	USA	301	TN	I-II	NSCLC	TIL	RFS	H&E	surgery
Hee Eun Lee[61]	2020	USA	120	TN	I-IV	NSCLC	CD20+	OS	IHC	surgery
Jianqing Hao[62]	2020	China	192	TN	I-IV	NSCLC	CD8+/Foxp3+	OS	IHC	surgery
Senga K. Johnson[63]	2000	Scotland	95	TN	I-III	NSCLC	CD3+/CD8+/CD57+/CD68+	OS	H&E/IHC	surgery
Katsuhiko Shimizu[64]	2010	Japan	100	TN	I-III	NSCLC	Foxp3+	RFS	IHC	surgery

TILs (tumour-infiltrating lymphocytes), TN (tumour nest), TS (tumour stroma), NSCLC (non-small cell lung cancer), FoxP3+ (factor forkhead box P3+), OS (overall survival), PFS (progression-free survival), DFS (disease-free survival), RFS (relapse/recurrence free survival), DSS (disease-specific survival), IHC (immunohistochemistry), H&E (haematoxylin and eosin).

### Summary of the quality and risk of bias of the included studies

We performed quality evaluations of the 45 articles following the QUIPS tool, and two authors independently evaluated all the literature. Differences were resolved by discussion. After screening all included articles, we found that no studies reported study attrition, and there was no standardization of the cut-off value for evaluating TIL expression. Thus, 24 studies were evaluated as low risk, 14 were evaluated as moderate risk, and 7 were evaluated as high risk (**S2 Table**). This outcome indicated that most of the studies we included were of medium or high quality.

### TILs as prognostic biomarkers

**Fig 2:**
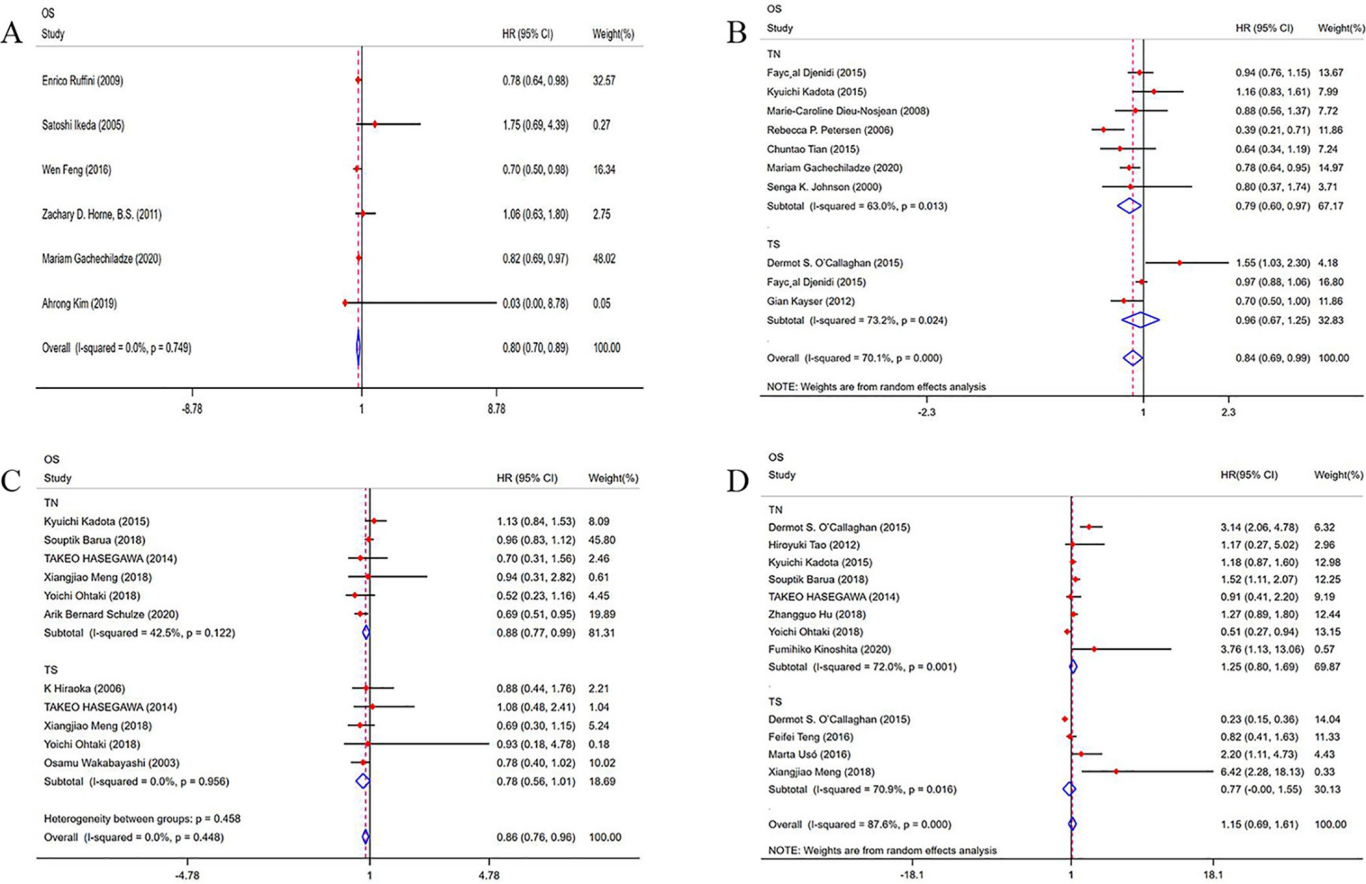
Forest plots of the prognostic value of TILs subtypes on overall survival in patients with NSCLC. TILs (tumour-infiltrating lymphocytes), OS (overall survival), FoxP3+ (factor forkhead box P3+), HRs (hazard ratios), 95% CIs (95% confidence intervals), TN (tumour nest), TS (tumour stroma). (A) Forest plots of the prognostic value of TIL; (B) Forest plots of the prognostic value of CD3+ TILs; (C) Forest plots of the prognostic value of CD4+ TILs; (D) Forest plots of the prognostic value of FoxP3+ TILs.

The prognostic value of TILs was assessed in 6 studies in our meta-analysis. As shown in **Figs 2A and** 6 studies were included in the TN group, and the pooled result showed that a high density of TILs in the TN indicated a better OS (HR = 0.80; 95% CI, 0.70–0.89; *p*<0.001) and PFS (HR = 0.73; 95% CI, 0.61–0.85; *p*<0.001) for patients with NSCLC (**S1 Fig**). However, no study analysed the relationship between TILs and RFS, DFS and DSS.

### CD3+ TILs as prognostic biomarkers

**Fig 3:**
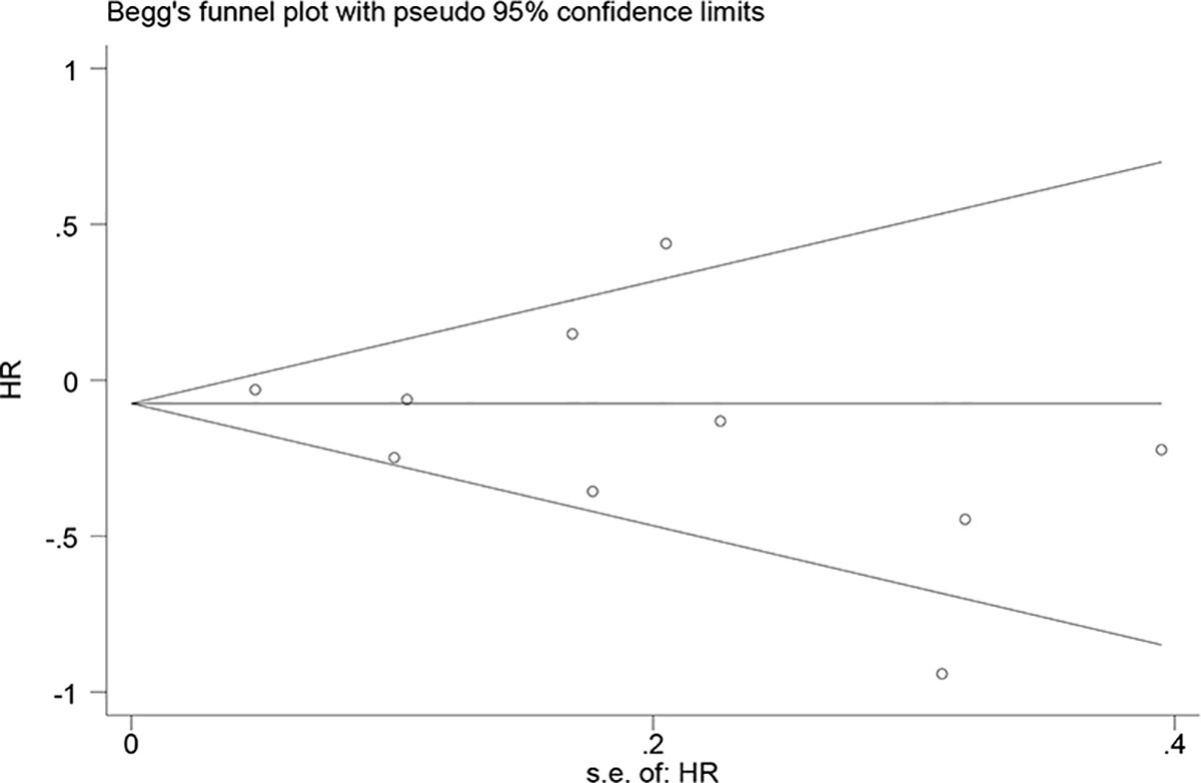
Funnel plot of CD3+ TILs on overall survival. TILs (tumour-infiltrating lymphocytes), HR (hazard ratios).

As shown in **Fig 2B and** 9 eligible articles were pooled for analysing the prognostic value of CD3+ TILs in NSCLC. Seven studies were included in the TN group, and 3 studies were included in the TS group, The results showed that a high density of CD3+ TILs in the TN indicated a better OS (HR = 0.79; 95% CI, 0.60–0.97; *p*<0.001) for patients with NSCLC, and a high density of CD3+ TILs in the TS indicated a better DSS (HR = 0.52; 95% CI, 0.27–0.77; *p*<0.001) for patients with NSCLC (**S2 Fig**). However, we observed no obvious favourable OS (HR = 0.96; 95% CI, 0.67–1.25; *p*<0.001) in the TS of patients with NSCLC. Begg’s test (*p* = 0.421) and Egger’s test (*p* = 0.240) results provided no evidence of publication bias. (**Table 2, Fig 3)**

**Table 2:** Summary of the prognostic value of different TIL subtypes in different locations of NSCLC.

TIL phenotypes	location	OS						DFS				DSS				RFS				PFS			
		No. of studies	*I*^*2*^ (*p*)	HR (95% CI)	*p* value	Begg's test(*p*)	Egger’s test(*p*)	No. of studies	*I*^*2*^ (*p*)	HR (95% CI)	*p* value	No. of studies	*I*^*2*^ (*p*)	HR (95% CI)	*p *value	No. of studies	*I*^*2*^ (*p*)	HR (95% CI)	*p* value	No. of studies	*I*^*2 *^(*p*)	HR (95% CI)	*p* value
TILs	TN	6	0%(0.749)	0.80(0.70,0.89)	<0.001															3	46.2%(0.156)	0.73(0.61,0.85)	<0.001
CD3+TILs	TN	7	63.0%(0.013)	0.79(0.60,0.97)	<0.001			2	0%(0.51)	0.84(0.65,1.02)	<0.001	1		0.99(0.29,1.69)	0.006								
	TS	3	73.2%(0.024)	0.96(0.67,1.25)	<0.001			1		0.96(0.88,1.04)	<0.001	1		0.52(0.27,0.77)	<0.001								
	overall	9	70.1%(<0.001)	0.84(0.69,0.99)	<0.001	0.421	0.240	2	0%(0.39)	0.94(0.87,1.01)	<0.001	2	34.5%(0.217)	0.57(0.34,0.80)	<0.001								
CD4+TILs	TN	6	42.5%(0.122)	0.88(0.77,0.99)	<0.001							1		0.75(0.52,0.98)	<0.001	2	0%(0.448)	0.67(0.29,1.05)	0.001				
	TS	5	0%(0.956)	0.78(0.56,1.01)	<0.001							2	0%(0.615)	0.45(0.30,0.59)	<0.001	2	0%(0.499)	1.0(0.26,1.74)	0.008				
	overall	8	0%(0.448)	0.86(0.76,0.96)	<0.001	0.697	0.239					2	60.1%(0.082)	0.56(0.35,0.78)	0.076	2	0%(0.647)	0.74(0.40,1.08)	<0.001				
CD8+TILs	TN	23	87.2%(<0.001)	0.98(0.97,1.0)	<0.001			3	0%(0.833)	0.74(0.57,0.91)	<0.001	1		0.75(0.52,0.98)	<0.001	2	0%(0.364)	0.37(0.10,0.65)	0.008	4	74.6%(0.008)	0.55(0.34,0.76)	<0.001
	TS	13	86.2%(<0.001)	0.997(0.991,1.0)	<0.001			3	90.3%(<0.001)	0.54(0.17,0.92)	0.004	2	0%(0.615)	0.45(0.30,0.59)	<0.001	1		0.59(0.07,1.11)	0.025	1		0.40(0.10,0.69)	0.008
	overall	27	86.5%(<0.001)	0.995(0.99,1.0)	<0.001	0.081	0.834	5	77.3%(0.001)	0.64(0.43,0.85)	<0.001	2	0%(0.391)	0.56(0.35,0.78)	<0.001	2	0%(0.511)	0.42(0.18,0.67)	0.001	5	69.9%(0.01)	0.52(0.34,0.71)	<0.001
FoxP3+TILs	TN	8	72.0%(0.001)	1.25(0.80,1.69)	<0.001			2	0%(0.349)	1.20(0.85,1.56)	<0.001					4	31.5%(0.223)	0.92(0.26,1.57)	0.006				
	TS	4	70.9%(0.016)	0.77(0.00,1.55)	0.051			1		1.30(0.50,2.10)	0.001					2	0%(0.743)	1.90(1.05,2.76)	<0.001				
	overall	11	87.6%(<0.001)	1.15(0.69,1.61)	<0.001	0.55	0.679	3	0%(0.630)	1.22(0.89,1.55)	<0.001					5	58.3%(0.035)	1.33(0.61,2.05)	<0.001				
CD20+TILs	TN	4	80.1%(0.002)	0.65(0.36,0.94)	<0.001							2	80.4%(0.024)	0.62(0.04,1.19)	0.035								

TILs (tumour-infiltrating lymphocytes), TN (tumour nest), TS (tumour stroma), NSCLC (non-small cell lung cancer), FoxP3+ (factor forkhead box P3+), OS (overall survival), PFS (progression-free survival), DFS (disease-free survival), RFS (relapse/recurrence free survival), DSS (disease-specific survival), HRs (hazard ratios), 95% CIs (95% confidence intervals)

### CD4+ TILs as prognostic biomarkers

We pooled 8 included articles to analyse the prognostic value of CD4+ TILs in NSCLC. Six studies were included in the TN group, and 5 studies were included in the TS group. The results showed that a high density of CD4+ TILs in the TN indicated a better OS (HR = 0.88; 95% CI, 0.77–0.99; *p*<0.001) and DSS (HR = 0.75; 95% CI, 0.52–0.98; *p*<0.001) for patients with NSCLC. We also observed that a high density of CD4+ TILs in TS indicated a better DSS (HR = 0.45; 95% CI, 0.30–0.59; *p*<0.001) (**Fig 2C, S3 Fig**). Begg’s test (*p* = 0.697) and Egger’s test (*p* = 0.239) results provided no evidence of publication bias (**Table 2, S4 Fig**). There were no studies that reported the relationship between CD4+ TILs and DFS and PFS.

### CD8+ TILs as prognostic biomarkers

**Fig 4:**
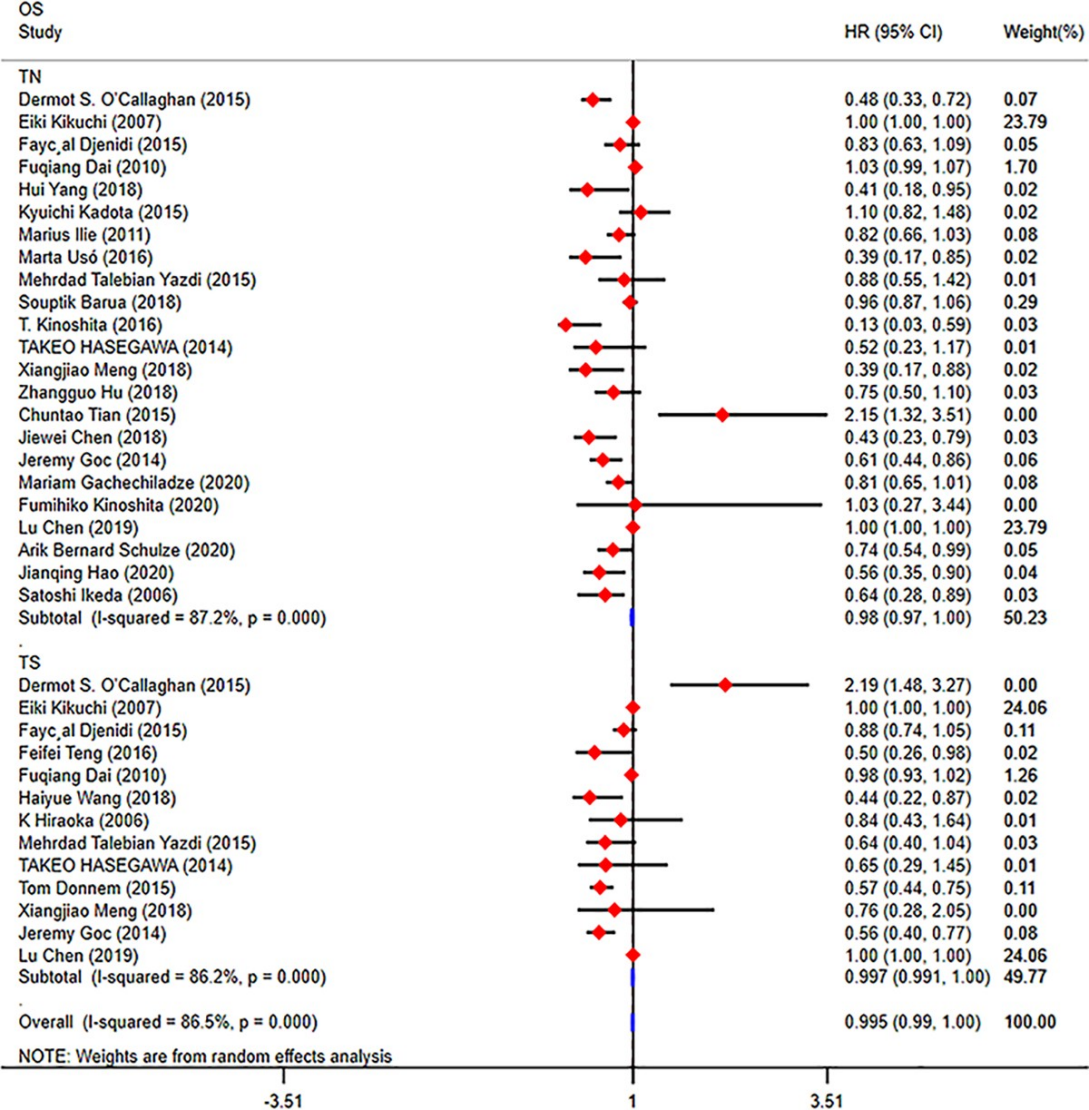
Forest plots of the prognostic value of CD8+ TILs on OS in patients with NSCLC. TILs (tumour-infiltrating lymphocytes), OS (overall survival), HRs (hazard ratios), 95% CIs (95% confidence intervals), TN (tumour nest), TS (tumour stroma).

Most previous studies reported the prognostic role of CD8+ TILs in NSCLC. As shown in **Fig 4,** we included 27 eligible articles for analysing the prognostic value of CD8+ TILs. Twenty-three studies were included in the TN group, and 13 studies were included in the TS group. The results showed that a high density of CD8+ TILs in the TN indicated a better OS (HR = 0.98; 95% CI, 0.97–1.0; *p*<0.001), PFS (HR = 0.55; 95% CI, 0.34–0.76; *p*<0.001), DFS (HR = 0.74; 95% CI, 0.57–0.91; *p*<0.001), RFS (HR = 0.37; 95% CI, 0.10–0.65; *p* = 0.008) and DSS (HR = 0.75; 95% CI, 0.52–0.98; *p*<0.001) for patients with NSCLC. In TS, a high density of CD8+ TILs also indicated a better OS (HR = 0.997; 95% CI, 0.99–1.0; *p*<0.001), PFS (HR = 0.40; 95% CI, 0.10–0.69; *p* = 0.008), DFS (HR = 0.54; 95% CI, 0.17–0.92; *p* = 0.004) and DSS (HR = 0.45; 95% CI, 0.30–0.59; *p*<0.001) for patients with NSCLC (**S5 Fig**). Begg’s test (*p* = 0.081) and Egger’s test (*p* = 0.834) results provided no evidence of publication bias. (**Table 2, S6 Fig**)

**Fig 5:**
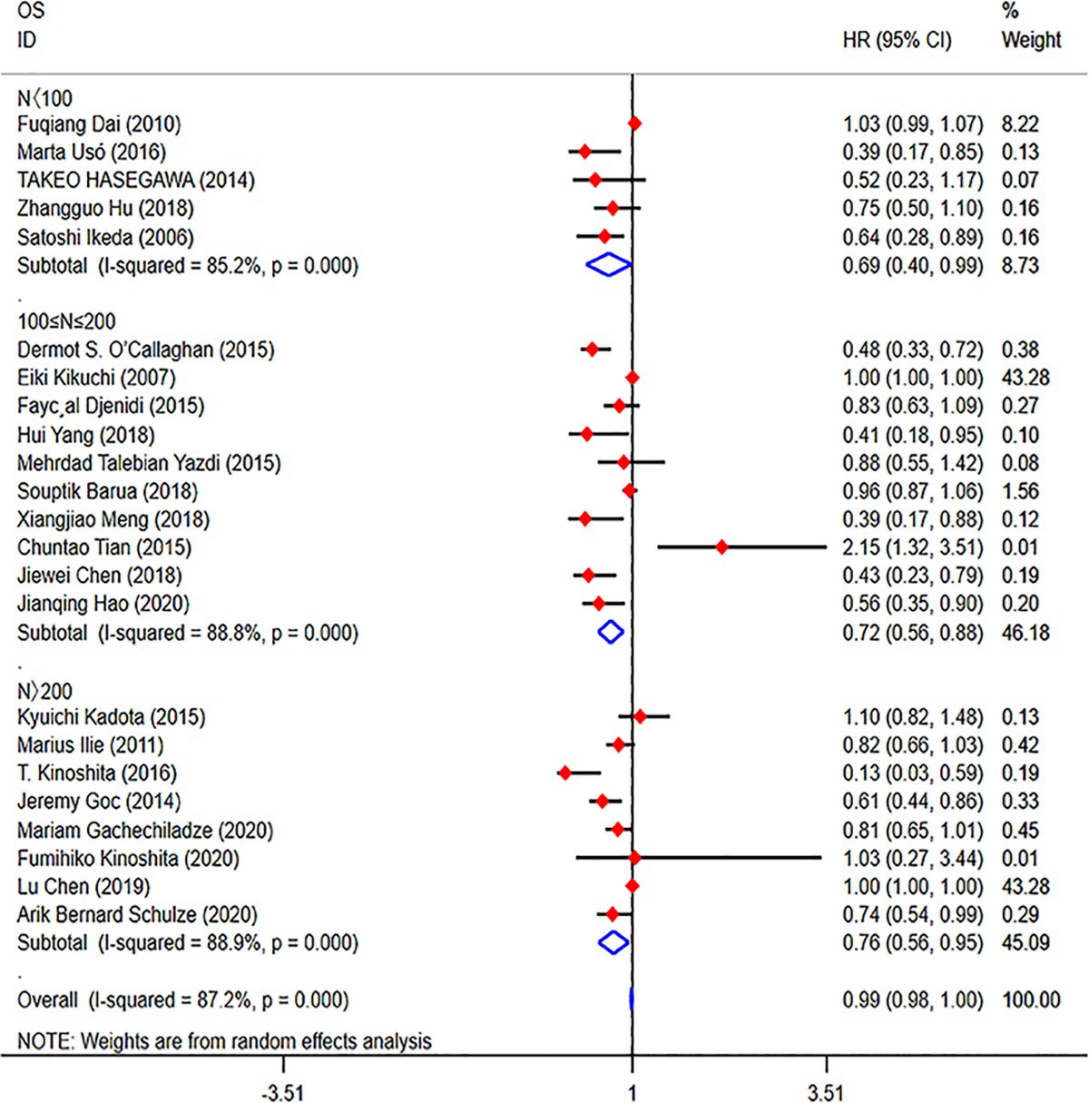
Forest plots of the subgroup analysis of CD8+ TILs in patients with NSCLC. TILs (tumour-infiltrating lymphocytes), OS (overall survival), HRs (hazard ratios), 95% CIs (95% confidence intervals).

We divided the 23 studies from the TN group into three groups according to the number of samples (N<100, 100≤N≤200, and N>200). Then, we performed a subgroup analysis, and we found that a high density of CD8+ TILs indicated a better OS (N<100: HR = 0.69; 95% CI, 0.40–0.99; *p*<0.001; 100≤N≤200: HR = 0.72; 95% CI, 0.56–0.88; p<0.001; N>200: HR = 0.76; 95% CI, 0.56–0.95; *p*<0.001) in the different groups (**Fig 5**). Because few studies have focused on the correlation between the prognostic value of TILs and tumour stage and histologic subtype, we failed to perform another subgroup analysis according to tumour stage and histologic subtype.

### FoxP3+ TILs as prognostic biomarkers

Eleven eligible articles were pooled to analyse the prognostic value of FoxP3+ TILs in NSCLC. As shown in **Fig 2D,** eight studies were included in the TN group, and 4 studies were included in the TS group. The results showed that a high density of Foxp3+ TILs in the TS indicated a worse RFS (HR = 1.90; 95% CI, 1.05–2.76; *p*<0.001) (**S7 Fig**). In the TN, there was no relationship between Foxp3+ TILs expression and prognosis for patients with NSCLC. Begg’s test (P = 0.55) and Egger’s test (P = 0.679) results provided no evidence of publication bias (**Table 2**).

### CD20+ TILs as prognostic biomarkers

**Fig 6:**
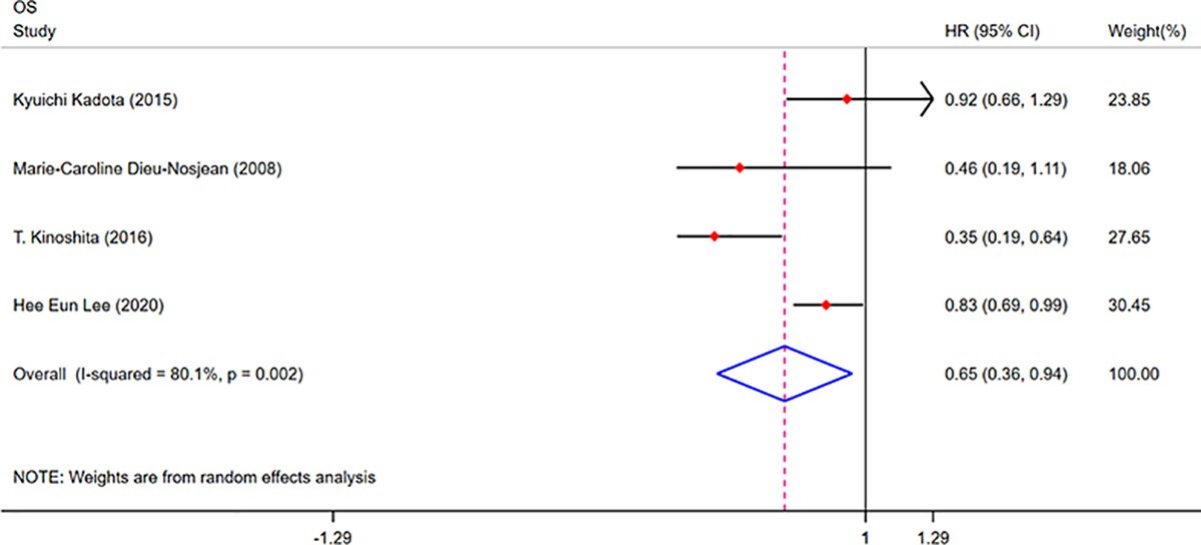
Forest plots of the prognostic value of CD20+ TILs on OS in patients with NSCLC. TILs (tumour-infiltrating lymphocytes), OS (overall survival), HRs (hazard ratios), 95% CIs (95% confidence intervals), TN (tumour nest), TS (tumour stroma).

We searched 4 studies to analyse the prognostic value of CD20+ TILs in NSCLC. Four studies were included in the TN group. As shown in **Fig 6,** the results showed that a high density of CD20+ TILs in the TN indicated a better OS (HR = 0.65; 95% CI, 0.36–0.94; *p*<0.001) for patients with NSCLC. However, we observed no better DSS for patients with NSCLC with a high density of CD20+ TILs, as shown in **S8 Fig.**

## Discussion

A large number of studies have shown that there was a significant correlation between TIL infiltration concentration and survival of patients with NSCLC, but the results of different studies remain controversial [34, 40, 20]. We included a total of 45 studies with 11,448 patients with NSCLC and provided quantitative estimates of the prognostic value of TILs in patients with NSCLC by pooling the HRs and 95% CIs.

Our meta-analysis indicated that a high density of TILs in the TN was correlated with better OS and PFS for patients with NSCLC. Many studies have demonstrated that a high density of TILs could prolong OS for patients with NSCLC [28, 30, 35, 65, 66]. Therefore, we can conclude a conclusion that TILs are a good prognostic factor for NSCLC, and the higher the density of TILs in the tumour nest, the better is the prognosis for patients with NSCLC. This outcome is supported by the study of Wen Feng [30]. The reason for this role of TILs in patients with NSCLC is that TILs act as an anti-tumour effector cells to kill abnormally proliferating lung cancer cells [67].

TILs consist of different subtypes, such as CD3+ TILs, CD4+ TILs, CD8+ TILs, CD20+ TILs and FoxP3+ TILs. The function of CD3+ TILs in patients with NSCLC is currently unclear. Petersen R P [36] reported that all patients could benefit from a high density of CD3+ TILs. However, other researchers held the opinion that high CD3+ TILs conferred worse survival for patients with NSCLC [20]. In our meta-analysis, 9 eligible articles were pooled to analyse the prognostic value of CD3+ TILs in NSCLC. The results showed that a high density of CD3+ TILs in the TN indicated a better OS for patients with NSCLC, and a high density of CD3+ TILs in the TS indicated a better DSS. The mechanism for this role of CD3+ TILs in patients with NSCLC is that CD3 is the most common phenotype of TILs, CD8+ TILs also express the CD3+ phenotype, and CD8+TILs are a good prognostic marker for NSCLC, Therefore, we hypothesize that mainly CD3+CD8+ T cells play a synergistic role in promoting the prognosis for patients with NSCLC. Our assumption is supported by Kayser G [49]. These outcomes were different from those of other studies [68, 69], and we considered that the reason for this difference might be caused by the number of included studies.

T cells present in tumour are mixtures of CD8+ T cells and CD4+ T cells. The proportion of CD4+ and CD8+ TILs in distinct tumour tissues is different, CD4+ TILs are related to humoral immunity, CD8+ TILs are related to cellular immunity, and CD8+ TILs play a major killing role in NSCLC [64, 65]. However, the main role of CD4+ T cells in the immune response to cancer is to prime CD8+ T cells and maintain their proliferation [65]. Eight included articles were pooled to analyse the prognostic value of CD4+ TILs in NSCLC. Overall, we observed that a high density of CD4+ TILs in the TN indicated a better OS and DSS for patients with NSCLC. In the TS, a high density of CD4+ TILs indicated a better DSS, but there was no relationship between CD4+ TILs and OS for patients with NSCLC. We obtained the opposite result of that from other studies [68, 69]. The explanation for this controversy may be that CD4+ TILs are related to humoral immunity and play a major role in the tumour nest. Moreover, after CD4+ T cells are activated, cytotoxic T lymphocytes (CTLs) can be activated by activated CD4+ T cells through various mechanisms to maintain and enhance the anti-tumor response of CTLs in tumours [70, 71]. On the other hand, regulatory T cells (Tregs) are a subtype of CD4+ T cells with immunosuppressive properties that express high levels of CD25 on their cell surface [49]. Therefore, CD4+ TILs in the TS may have the opposite effect on tumour immunity, and many previous studies could support this conclusion [72, 73]. Thus, the favourable role of CD4+ TILs in patients with NSCLC is probably closely related to the abovementioned functions of CD4+ TILs.

We included 27 eligible articles and proved that a high density of CD8+ TILs was related to a favourable prognostic role for patients with NSCLC. The results showed that a high density of CD8+ TILs in the TN indicated a better OS, PFS, DFS, RFS and DSS for patients with NSCLC, as well as a better OS, PFS, DFS and DSS in the TS. Our results showed that CD8+ TILs play an important role in the TN and TS in NSCLC. This may be closely related to the direct killing effect of CD8+ TILs on tumour cells. Our results were consistent with those of previous studies [68, 69], and the results were credible. Although we performed a subgroup analysis according to the number of the sample, we did not find the source of heterogeneity. We thought that the heterogeneity might be related to the detection method of TILs. Due to the prognostic value of CD8+ TILs in both the TN and TS, some studies have indicated that patients with surgically resected stage I NSCLC showing a low density of CD8+ TILs could be considered for adjuvant chemotherapy, even if they have no high-risk features [51].

Many studies have indicated that FoxP3+ TILs are associated with a good prognosis [16, 32], while others envisage that FoxP3+ TILs are a poor prognosis factor for NSCLC [34, 39]. There is no unified conclusion about the controversies. Eleven eligible articles were included in our meta-analysis to analyse the prognostic value of FoxP3+ TILs in NSCLC. Overall, the survival of patients with NSCLC is not connected with the density of FoxP3+ TILs. However, for the subgroup analysis, the results demonstrated that a high density of FoxP3+ TILs in the TN indicated a worse RFS for patients with NSCLC. And no significant difference was found for OS or DFS in the TN or TS. Our results demonstrated that FoxP3+ TILs may be associated with tumour recurrence. Many studies have confirmed the results that CD25 and the transcription factor FoxP3 are highly expressed in Treg cells and that FoxP3 is a transcriptional repressor required for maturation and immunosuppressive functionality [74, 75]. A high density of FoxP3+ TILs in the TN indicated an immunosuppressive environment, so it was the reason for tumour recurrence. The results are inconsistent with other studies. Dong-Qiang Zeng reported that a low density of FoxP3+ regulatory TILs was found to correlate with a good overall or recurrence-free survival [68], and another meta-analysis showed that a high density of FoxP3+ TILs in the TS is associated with poor outcome in NSCLC [69]. The possible explanation for the inconsistent role of FoxP3+ TILs is that FoxP3 is not specific for activated Tregs, and it is necessary to conjointly assess FoxP3 and additional markers, such as CD4, CD8 and CD25 [76, 77]. Nevertheless, few studies have assessed this combination in NSCLC.

CD20 is expressed on the surface of B cells, and plays an important regulatory role in the proliferation and differentiation of B cells. CD20 is also a phenotype of TILs, and the prognostic role of CD20+ TILs has rarely been assessed in various tumours. We searched 4 studies to analyse the prognostic value of CD20+ TILs in NSCLC. We observed that a high density of CD20+ TILs in the TN indicated a better OS for patients with NSCLC. Our results showed that humoral immunity played an equally important role as cellular immunity in the tumour nest. Our meta-analysis is the first study to evaluate the value of CD20+ TILs in NSCLC, but large-sample studies are needed to verify the results on account of the small number of included studies.

Although, our meta-analysis was not the first study to focus on the correlation between the density of TILs and prognosis. However, we drew a conclusion that is inconsistent with previous studies by including 45 eligible articles and 11,448 patients. Our results demonstrated that a high density of TILs, CD3+ TILs, CD4+ TILs, CD8+ TILs and CD20+ TILs in the TN are favourable prognostic biomarkers for patients with NSCLC, and a high density of Foxp3+ TILs in the TN is a poor prognostic biomarker. We found that TILs mainly played a prognostic role in the TN of patients with NSCLC, but in the TS, only CD8+ TILs played an important role in patients with NSCLC. Our results demonstrated that different TIL subtypes played a different prognostic role in the TN or TS in patients with NSCLC. Therefore, the density of TILs can be detected through cancer tissues and used as an indicator for treating NSCLC, evaluating prognosis and monitoring recurrence or metastasis in the future.

Although our study fully explains the relationship between the subtypes of TILs and survival of patients with NSCLC, several limitations of our meta-analysis should be mentioned. First, the great heterogeneity can be observed in most of the pooled results, The explanation for this situation is that the evaluation criteria for the density of TILs are particularly mixed, and there are no international standards exists on cut-off values to evaluate the density of TILs. Thus, the scoring methods and cut-off values of TILs should be unified to strengthen the conclusions on prognostic biomarkers of TILs. Otherwise, the detection method of TILs in most studies is mainly immunohistochemistry at present, which is a semiquantitative, subjective and inaccurate detection method. Therefore, different studies show different prognostic values of TILs. We thus need another more precise detection method to evaluate the expression of TILs in NSCLC in the future. Moreover, most studies evaluated the density of TILs by pathologists readings, and this evaluation method may produce errors. Therefore, a study from Germán Corredor presented a new set of computer-extracted quantitative features (SpaTIL) related to the spatial architecture of TILs, the colocalization of TILs and cancer nuclei, and the density variation of TIL clusters from H&E images [12]. This new method can effectively reduce the human error.

Second, more high-quality studies on the relationship between tumour stage, histologic subtype, patients’ age at NSCLC diagnosis and the density of TILs need to be focused on to verify our results. Otherwise, the therapy method is also a key limitation. The current studies only focused on patients with NSCLC after surgery, and very few studies mentioned other therapy methods in their reports. However, the prognostic value of TILs may possibly to lie in the therapeutic method. Therefore, different therapy methods for each patients with NSCLC should be focused on. Furthermore, TILs also included B cells, DCs, NK cells and other immune cell types, but few studies focused on this field. So it is important to explore the function of other immune cell types in NSCLC.

Third, some of the original studies did not report data about HRs and 95% CIs, and the HRs and 95% CIs results were extracted from Kaplan–Meier survival curves, which introduces certain deviation and subjectivity. Therefore, the authenticity of the results might be influenced by this approach.

## Conclusion

In summary, our meta-analysis confirmed that high densities of TILs, CD3+ TILs, CD4+ TILs, CD8+ TILs and CD20+ TILs are favourable prognostic biomarkers for patients with NSCLC, and Foxp3+ TILs are a poor prognostic biomarker. Thus, TILs have shown prognostic value for patients with NSCLC, and detecting the density of TILs in pathological diagnosis will be helpful to guide the treatment and prognosis for patients with NSCLC. For our research, other high-quality studies are required to confirm our findings about the prognostic value of TILs in NSCLC in the future. In view of the limitations of our analysis, the conclusions should be interpreted with caution.

## Supporting information

S1 Table The PRISMA checklist. (PDF)

S2 Table Quality assessment of included studies. (PDF)

S1 Fig Forest plots of the prognostic value of TIL on PFS in patients with NSCLC. (TIF)

S2 Fig Forest plots of the prognostic value of CD3+ TIL on DFS and DSS in patients with NSCLC. (TIF)

S3 Fig Forest plots of the prognostic value of CD4+ TIL on DSS and RFS in patients with NSCLC. (TIF)

S4 Fig Funnel plot of CD4+ TIL on overall survival. (TIF)

S5 Fig Forest plots of the prognostic value of CD8+ TIL in patients with NSCLC. (TIF)

S6 Fig Funnel plot of CD8+ TIL on overall survival. (TIF)

S7 Fig Forest plots of the prognostic value of FoxP3+ TIL on DFS and RFS in patients with NSCLC. (TIF)

S8 Fig Forest plots of the prognostic value of CD20+ TIL on DSS in patients with NSCLC. (TIF)

S1 File The list of search strategy. (PDF)
