# Supplementary material for: Prognostic value of the common tumour-infiltrating lymphocyte subtypes for patients with non-small cell lung cancer: A meta-analysis
Source: PLoS One. 2020 Nov 10;15(11):e0242173. doi: 10.1371/journal.pone.0242173 (PMC7654825; doi:10.1371/journal.pone.0242173)
Supplement: S2 Table — (PDF) [file pone.0242173.s002.pdf]

S2 Table. Quality assessment of included studies.○= low risk of bias, ⦿= moderate risk of bias,●= high risk of bias.

Study	Study Participation	Study Attrition	Prognostic factor Measurement	Outcome Measurement	Study Confounding	Statistical Analysis and Reporting	Total Risk of Bias
Jeremy Goc	○	●	⦿	⦿	○	○	Moderate
Jiewei Chen	○	●	○	○	○	○	Low
Yoichi Ohtaki	○	⦿	○	○	○	○	Low
Chuntao Tian	○	●	⦿	○	○	○	Low
Zhangguo Hu	○	●	⦿	○	⦿	○	Moderate
Zachary D. Horne, B.S.	⦿	●	⦿	⦿	⦿	○	High
Xiangjiao Meng	○	⦿	○	○	○	○	Low
Wen Feng	○	●	⦿	○	○	○	Low
Tom Donnem	○	●	⦿	○	⦿	○	Moderate
TAKEO HASEGAWA	○	●	○	○	○	○	Low
T. Kinoshita	○	●	⦿	○	⦿	○	Moderate

Souptik Barua	○	◉	○	○	○	○	Low
Satoshi Ikeda	○	●	◉	◉	◉	○	High
Rebecca P. Petersen	○	●	◉	◉	○	○	Moderate
Osamu Wakabayashi	○	●	◉	◉	○	○	Moderate
Mehrdad Talebian Yazdi	◉	●	●	◉	○	○	High
Marta Usó	◉	●	◉	○	●	○	High
Marius Ilie	○	●	◉	◉	○	○	Moderate
Marie-Caroline Dieu-Nosjean	○	●	○	○	◉	○	Low
Kyuichi Kadota	○	◉	○	○	○	○	Low
Khalid I. Al-Shibli	○	●	○	○	◉	○	Low
KHALID AL-SHIBLI	○	●	○	○	◉	○	Low
K Hiraoka	○	●	◉	◉	○	○	Moderate
Hui Yang	○	◉	◉	○	○	○	Low

Hiroyuki Tao	○	●	⦿	⦿	○	○	Moderate
Haiyue Wang	○	●	⦿	⦿	⦿	○	High
Gian Kayser	○	●	○	○	○	○	Low
Fuqiang Dai	○	●	○	○	○	○	Low
Feifei Teng	⦿	●	⦿	○	⦿	○	High
Fayc¸al Djenidi	○	●	○	○	○	○	Low
Enrico Ruffini	○	●	⦿	○	⦿	○	Moderate
Eiki Kikuchi	○	●	⦿	○	○	○	Low
Dermot S. O’Callaghan	○	⦿	○	○	○	○	Low
Mariam Gachechiladze	○	⦿	○	○	○	○	Low
Fumihiko Kinoshita	○	●	○	○	○	○	Low
Ahrong Kim	○	●	⦿	⦿	○	○	Moderate
Lu Chen	⦿	●	⦿	○	○	○	Moderate

Yoshinori Handa	○	●	○	○	⊙	○	Low
Arik Bernard Schulze	○	●	⊙	○	⊙	○	Moderate
Kei Suzuki	○	●	⊙	⊙	○	○	Moderate
Germán Corredor	○	⊙	⊙	○	○	○	Low
Hee Eun Lee	○	●	⊙	○	○	○	Low
Jianqing Hao	○	●	○	○	○	○	Low
Senga K. Johnson	○	●	⊙	⊙	⊙	○	High
Katsuhiko Shimizu	○	●	⊙	○	○	○	Low
