# Supplementary figures and images for: Prognostic value of the common tumour-infiltrating lymphocyte subtypes for patients with non-small cell lung cancer: A meta-analysis
Source: PLoS One. 2020 Nov 10;15(11):e0242173. doi: 10.1371/journal.pone.0242173 (PMC7654825; doi:10.1371/journal.pone.0242173)

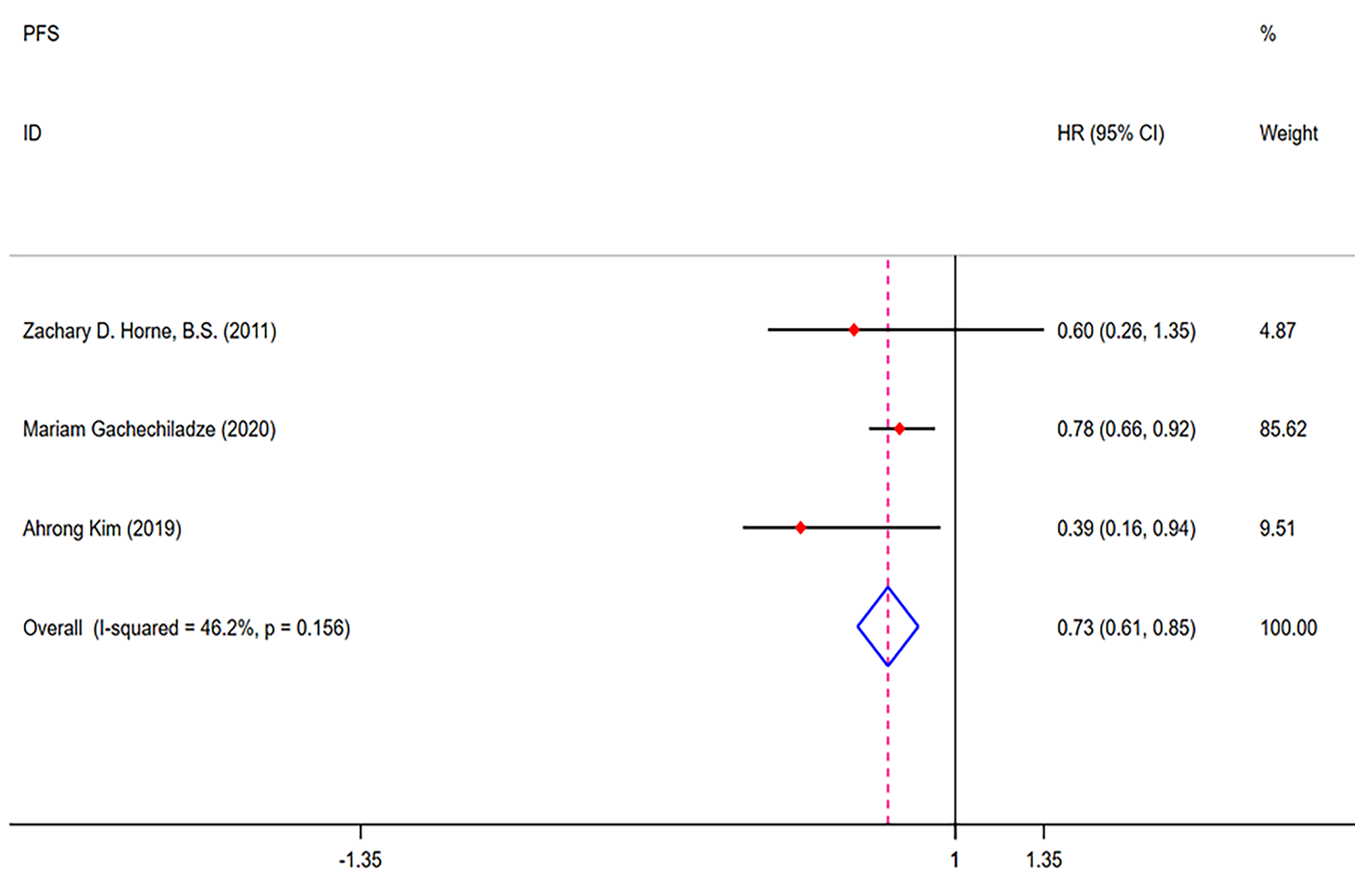

Supplement: S1 Fig — (TIF) [file pone.0242173.s003.tif]

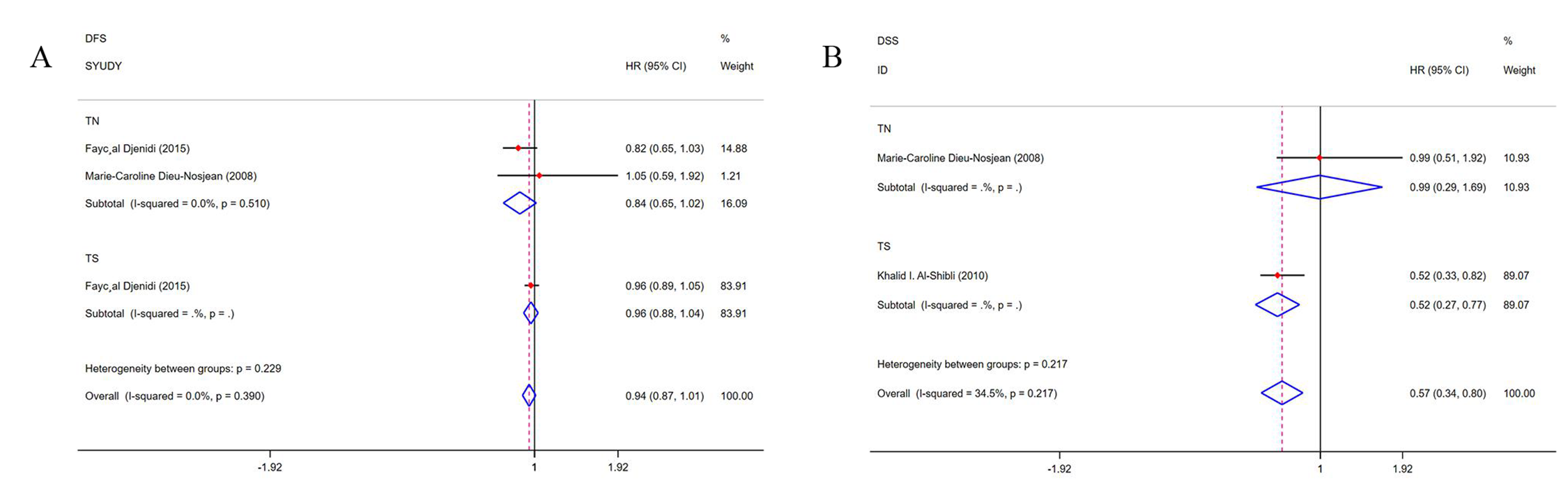

Supplement: S2 Fig — (TIF) [file pone.0242173.s004.tif]

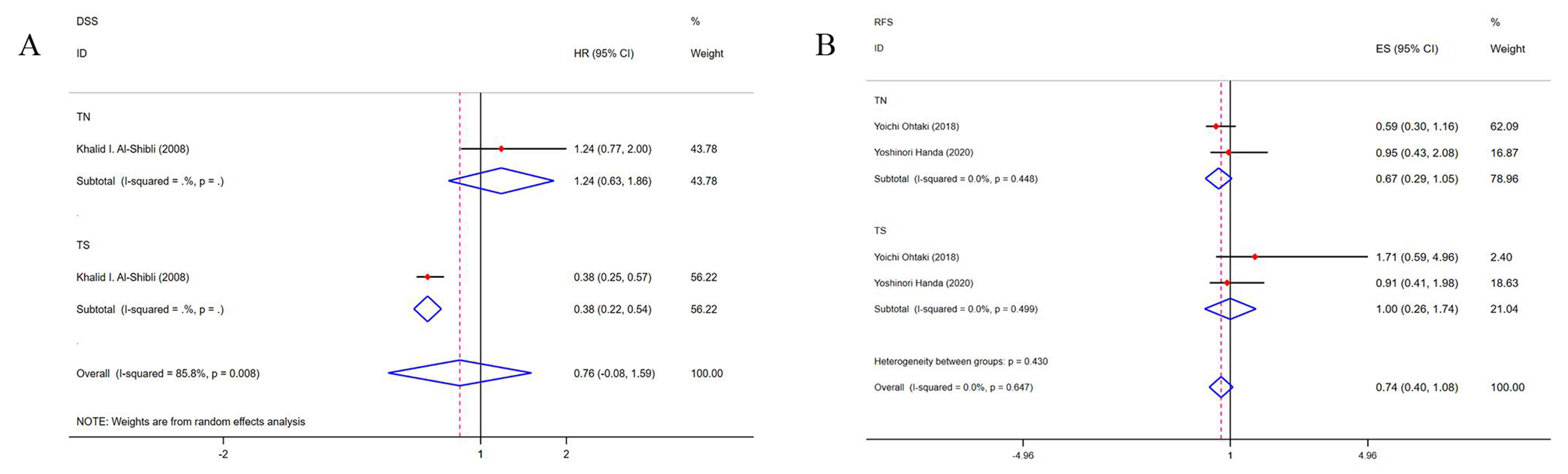

Supplement: S3 Fig — (TIF) [file pone.0242173.s005.tif]

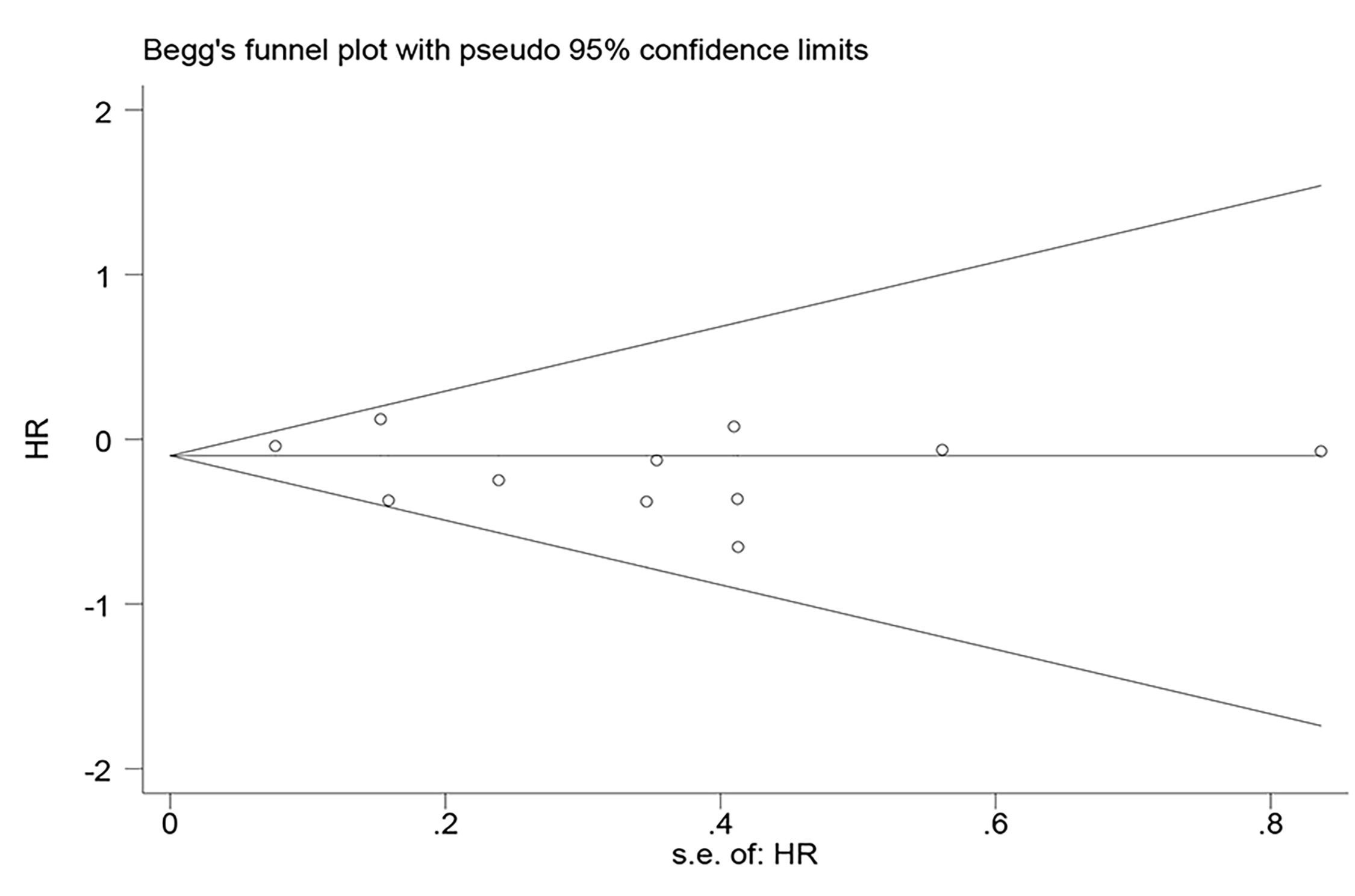

Supplement: S4 Fig — (TIF) [file pone.0242173.s006.tif]

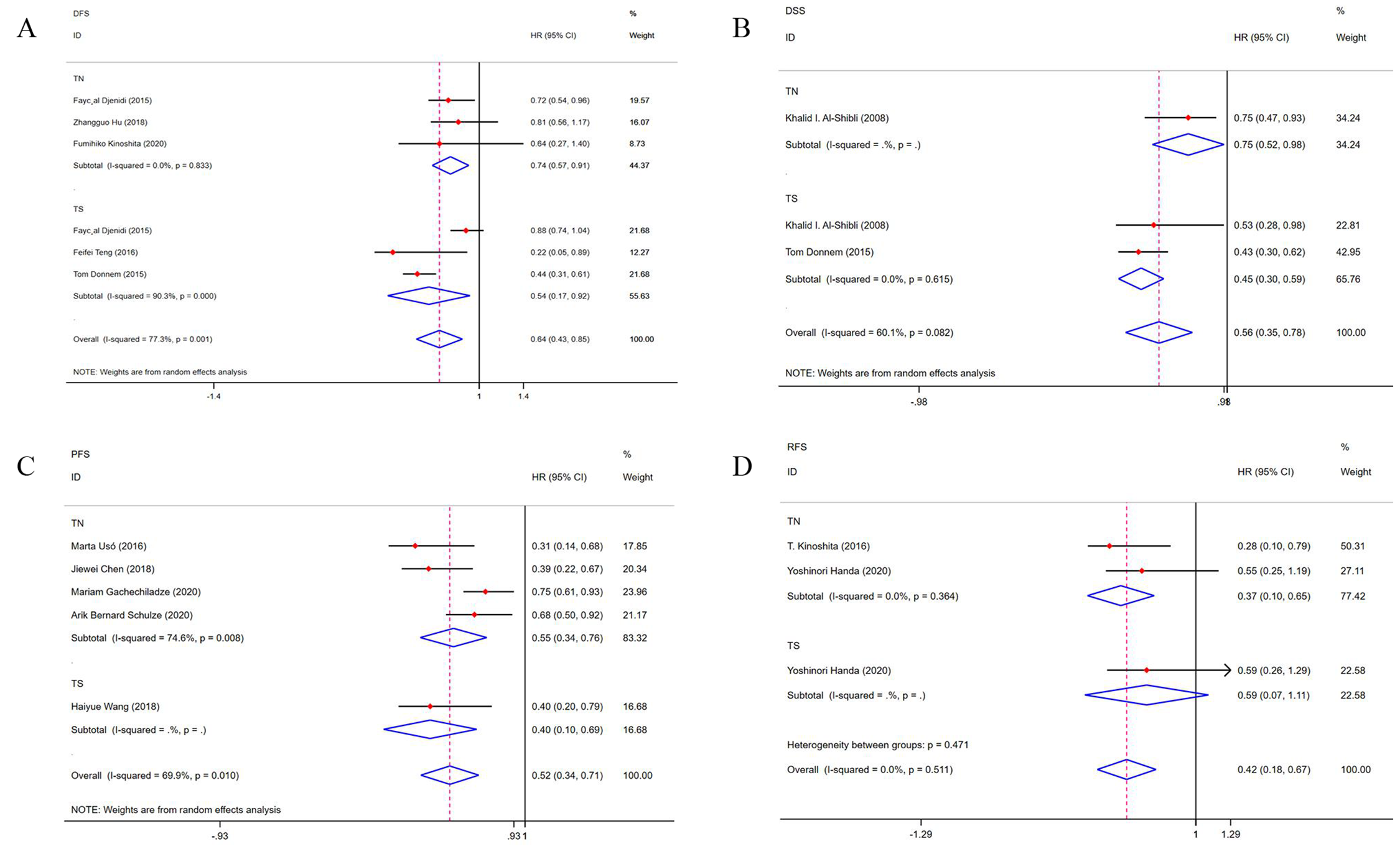

Supplement: S5 Fig — (TIF) [file pone.0242173.s007.tif]

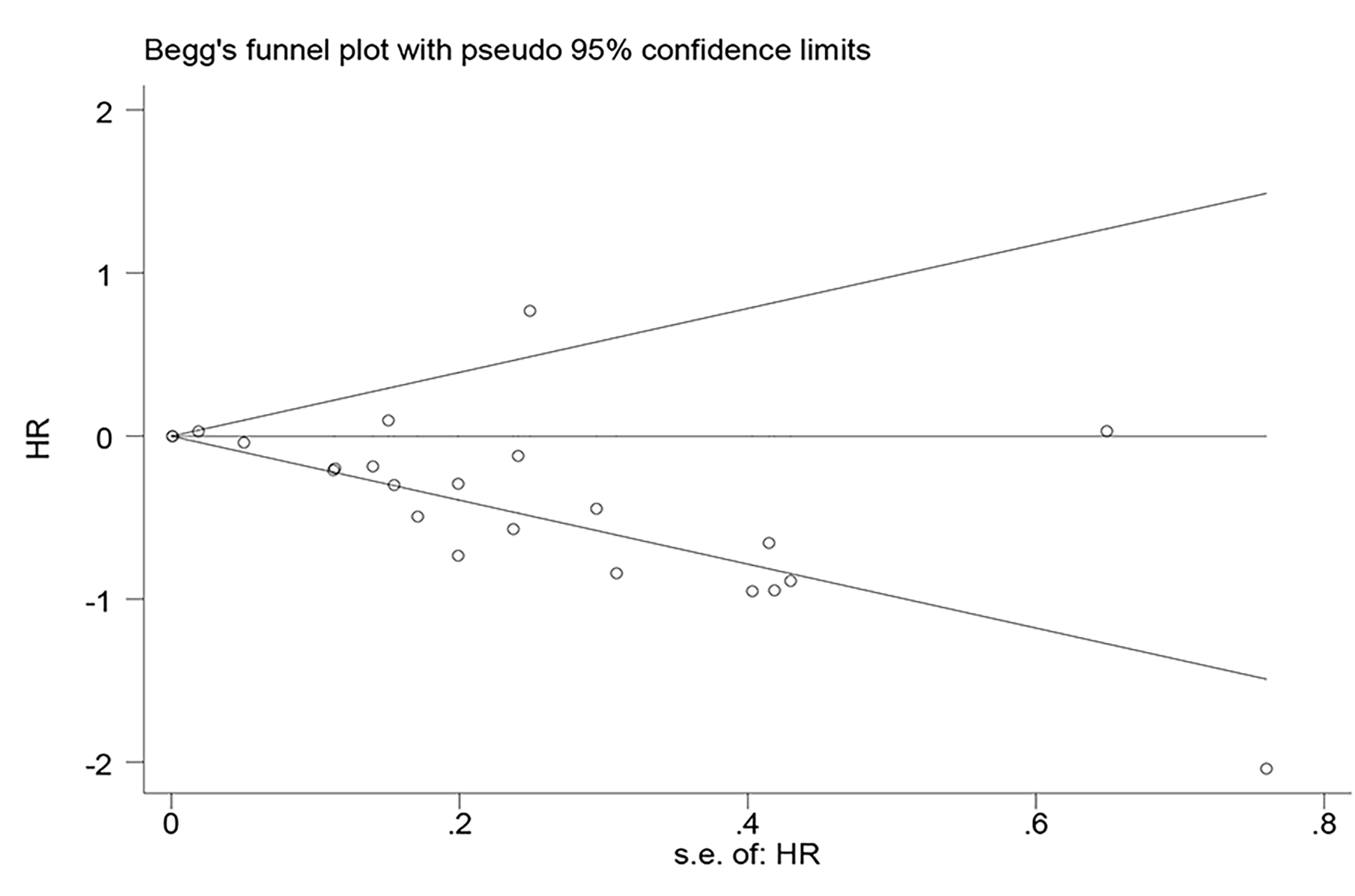

Supplement: S6 Fig — (TIF) [file pone.0242173.s008.tif]

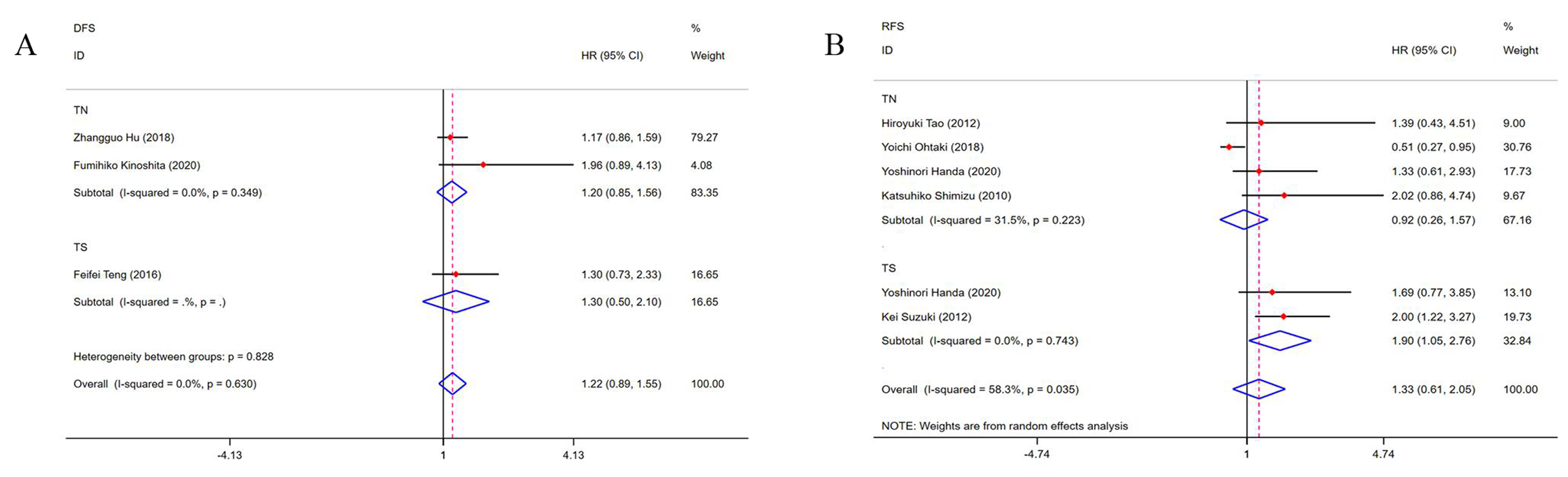

Supplement: S7 Fig — (TIF) [file pone.0242173.s009.tif]

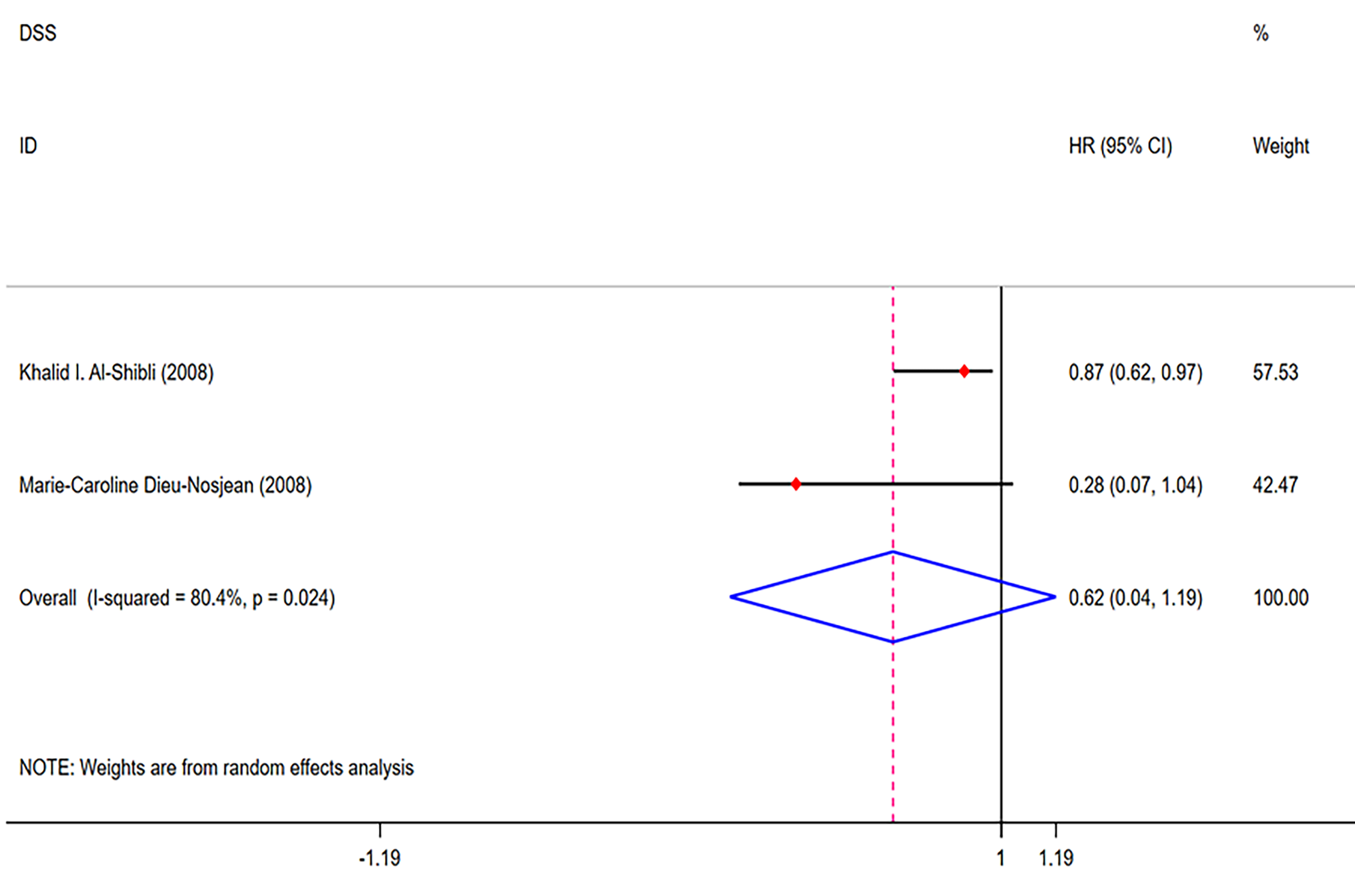

Supplement: S8 Fig — (TIF) [file pone.0242173.s010.tif]
