# Supplementary material for: Prognostic value of the common tumour-infiltrating lymphocyte subtypes for patients with non-small cell lung cancer: A meta-analysis
Source: PLoS One. 2020 Nov 10;15(11):e0242173. doi: 10.1371/journal.pone.0242173 (PMC7654825; doi:10.1371/journal.pone.0242173)
Supplement: S1 File — (PDF) [file pone.0242173.s011.pdf]

PubMed Advanced Search Builder

Add terms to the query box

Title/Abstract

Enter a search term

ADD

Show Index

Query box

Enter / edit your search query here

Search

History and Search Details

Search	Actions	Details	Query	Results	Time
#27	•••		Search: ((((((((((((tumor-Infiltrating lymphocyte[Title/Abstract]) OR (tumor-Infiltrating lymphocytes[Title/Abstract])) OR (tumor Infiltrating lymphocytes[Title/Abstract])) OR (tumor Infiltrating lymphocyte[Title/Abstract])) OR (TIL[Title/Abstract])) OR (TILs[Title/Abstract])) OR (intratumoral lymphocyte[Title/Abstract])) OR (intra-tumoral lymphocyte[Title/Abstract])) OR (FOXP3-positive T lymphocytes[Title/Abstract])) OR (CD8-positive T Lymphocytes[Title/Abstract])) OR (CD3-positive T Lymphocytes[Title/Abstract])) OR (CD4-positive T Lymphocytes[Title/Abstract])) OR (CD20-positive Lymphocytes[Title/Abstract])) AND ((((((((non-small cell lung cancer[Title/Abstract]) OR (NSCLC[Title/Abstract])) OR (small cell lung cancer[Title/Abstract])) OR (SCLC[Title/Abstract])) OR (Lung Squamous cell carcinoma[Title/Abstract])) OR (lung cancer[Title/Abstract])) OR (lung tumor[Title/Abstract])) OR (lung carcinoma[Title/Abstract])) OR (pulmonary[Title/Abstract]))	636	01:12:25
#25	•••		Search: ((((((((non-small cell lung cancer[Title/Abstract]) OR (NSCLC[Title/Abstract])) OR (small cell lung cancer[Title/Abstract])) OR (SCLC[Title/Abstract])) OR (Lung Squamous cell carcinoma[Title/Abstract])) OR (lung cancer[Title/Abstract])) OR (lung tumor[Title/Abstract])) OR (lung carcinoma[Title/Abstract])) OR (pulmonary[Title/Abstract])	705,234	01:11:45
#24	•••		Search: pulmonary[Title/Abstract]	546,874	01:11:25
#23	•••		Search: lung carcinoma[Title/Abstract]	18,533	01:11:15
#22	•••		Search: lung tumor[Title/Abstract]	6,241	01:11:03
#21	•••		Search: lung cancer[Title/Abstract]	157,613	01:10:41
#20	•••		Search: Lung Squamous cell carcinoma[Title/Abstract]	1,387	01:10:31
#19	•••		Search: SCLC[Title/Abstract]	7,894	01:10:21
#18	•••		Search: small cell lung cancer[Title/Abstract]	67,124	01:09:55
#17	•••		Search: NSCLC[Title/Abstract]	43,732	01:09:43
#16	•••		Search: non-small cell lung cancer[Title/Abstract]	57,264	01:09:31
#14	•••		Search: ((((((((((((tumor-Infiltrating lymphocyte[Title/Abstract]) OR (tumor-Infiltrating lymphocytes[Title/Abstract])) OR (tumor Infiltrating lymphocytes[Title/Abstract])) OR (tumor Infiltrating lymphocyte[Title/Abstract])) OR (TIL[Title/Abstract])) OR (TILs[Title/Abstract])) OR (intratumoral lymphocyte[Title/Abstract])) OR (intra-tumoral lymphocyte[Title/Abstract])) OR (FOXP3-positive T lymphocytes[Title/Abstract])) OR (CD8-positive T Lymphocytes[Title/Abstract])) OR (CD3-positive T Lymphocytes[Title/Abstract])) OR (CD4-positive T Lymphocytes[Title/Abstract])) OR (CD20-positive Lymphocytes[Title/Abstract])	7,507	01:08:41
#13	•••		Search: CD20-positive Lymphocytes[Title/Abstract]	23	01:07:35
#12	•••		Search: CD4-positive T Lymphocytes[Title/Abstract]	176	01:07:25
#11	•••		Search: CD3-positive T Lymphocytes[Title/Abstract]	90	01:07:15
#10	•••		Search: CD8-positive T Lymphocytes[Title/Abstract]	183	01:07:05
#9	•••		Search: FOXP3-positive T lymphocytes[Title/Abstract]	26	01:06:55
#8	•••		Search: intra-tumoral lymphocyte[Title/Abstract]	42	01:06:43
#7	•••		Search: intratumoral lymphocyte[Title/Abstract]	22	01:06:30
#6	•••		Search: TILs[Title/Abstract]	2,611	01:06:19
#5	•••		Search: TIL[Title/Abstract]	3,097	01:06:12
#4	•••		Search: tumor Infiltrating lymphocyte[Title/Abstract]	647	01:06:02
#3	•••		Search: tumor Infiltrating lymphocytes[Title/Abstract]	5,055	01:05:42
#2	•••		Search: tumor-Infiltrating lymphocytes[Title/Abstract]	5,055	01:05:22
#1	•••		Search: tumor-Infiltrating lymphocyte[Title/Abstract]	647	01:05:08

Showing 1 to 25 of 25 entries

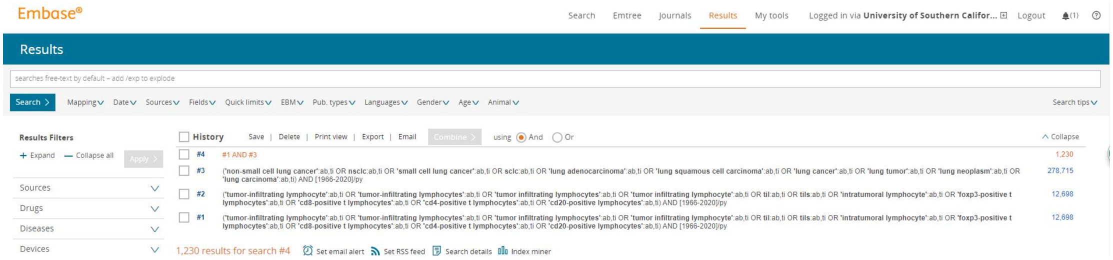

Embase®

Search | Emtree | Journals | Results | My tools | Logged in via **University of Southern Califor...** | Logout

Results

searches free-text by default – add /exp to explode

Search | Mapping | Date | Sources | Fields | Quick limits | EBM | Pub. types | Languages | Gender | Age | Animal | Search tips

Results Filters: Expand | Collapse all | Apply — Sources, Drugs, Diseases, Devices

History: Save | Delete | Print view | Export | Email | Combine | using And / Or | Collapse

Set	Query	Results
#4	#1 AND #3	1,230
#3	('non-small cell lung cancer':ab,ti OR nsclc:ab,ti OR 'small cell lung cancer':ab,ti OR sclc:ab,ti OR 'lung adenocarcinoma':ab,ti OR 'lung squamous cell carcinoma':ab,ti OR 'lung cancer':ab,ti OR 'lung tumor':ab,ti OR 'lung neoplasm':ab,ti OR 'lung carcinoma':ab,ti) AND [1966-2020]/py	278,715
#2	('tumor-infiltrating lymphocyte':ab,ti OR 'tumor-infiltrating lymphocytes':ab,ti OR 'tumor infiltrating lymphocytes':ab,ti OR 'tumor infiltrating lymphocyte':ab,ti OR til:ab,ti OR tils:ab,ti OR 'intratumoral lymphocyte':ab,ti OR 'foxp3-positive t lymphocytes':ab,ti OR 'cd8-positive t lymphocytes':ab,ti OR 'cd4-positive t lymphocytes':ab,ti OR 'cd20-positive lymphocytes':ab,ti) AND [1966-2020]/py	12,698
#1	('tumor-infiltrating lymphocyte':ab,ti OR 'tumor-infiltrating lymphocytes':ab,ti OR 'tumor infiltrating lymphocytes':ab,ti OR 'tumor infiltrating lymphocyte':ab,ti OR til:ab,ti OR tils:ab,ti OR 'intratumoral lymphocyte':ab,ti OR 'foxp3-positive t lymphocytes':ab,ti OR 'cd8-positive t lymphocytes':ab,ti OR 'cd4-positive t lymphocytes':ab,ti OR 'cd20-positive lymphocytes':ab,ti) AND [1966-2020]/py	12,698

1,230 results for search #4 | Set email alert | Set RSS feed | Search details | Index miner

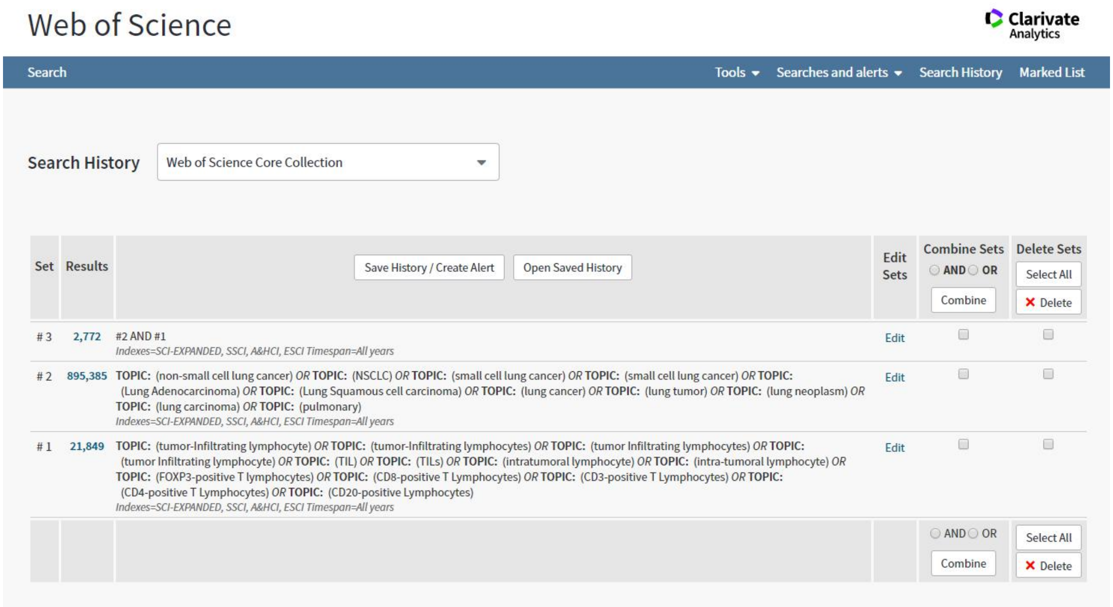

Web of Science

Clarivate Analytics

Search | Tools | Searches and alerts | Search History | Marked List

Search History

Web of Science Core Collection

Save History / Create Alert | Open Saved History

Set	Results	Query	Edit Sets
# 3	2,772	#2 AND #1 *Indexes=SCI-EXPANDED, SSCI, A&HCI, ESCI Timespan=All years*	Edit
# 2	895,385	**TOPIC:** (non-small cell lung cancer) *OR* **TOPIC:** (NSCLC) *OR* **TOPIC:** (small cell lung cancer) *OR* **TOPIC:** (small cell lung cancer) *OR* **TOPIC:** (Lung Adenocarcinoma) *OR* **TOPIC:** (Lung Squamous cell carcinoma) *OR* **TOPIC:** (lung cancer) *OR* **TOPIC:** (lung tumor) *OR* **TOPIC:** (lung neoplasm) *OR* **TOPIC:** (lung carcinoma) *OR* **TOPIC:** (pulmonary) *Indexes=SCI-EXPANDED, SSCI, A&HCI, ESCI Timespan=All years*	Edit
# 1	21,849	**TOPIC:** (tumor-Infiltrating lymphocyte) *OR* **TOPIC:** (tumor-Infiltrating lymphocytes) *OR* **TOPIC:** (tumor Infiltrating lymphocytes) *OR* **TOPIC:** (tumor Infiltrating lymphocyte) *OR* **TOPIC:** (TIL) *OR* **TOPIC:** (TILs) *OR* **TOPIC:** (intratumoral lymphocyte) *OR* **TOPIC:** (intra-tumoral lymphocyte) *OR* **TOPIC:** (FOXP3-positive T lymphocytes) *OR* **TOPIC:** (CD8-positive T Lymphocytes) *OR* **TOPIC:** (CD3-positive T Lymphocytes) *OR* **TOPIC:** (CD4-positive T Lymphocytes) *OR* **TOPIC:** (CD20-positive Lymphocytes) *Indexes=SCI-EXPANDED, SSCI, A&HCI, ESCI Timespan=All years*	Edit

Combine Sets: AND / OR — Combine | Delete Sets: Select All — Delete
